# Student use of common online resources in a multi-campus medical school

**DOI:** 10.15694/mep.2021.000056.1

**Published:** 2021-02-26

**Authors:** Steve Gallagher, Tehmina Gladman, Emma Macfarlane, Scott Hallman, John Hutton, Helen Paterson

**Affiliations:** 1 Otago Medical School - Dunedin Campus; 2 Otago Medical School - Wellington Campus; 3 Department of Women's and Children's Health; 4 Otago Medical School - Christchurch Campus; 5 Department of Obstetrics and Gynaecology

**Keywords:** elearning, online resources, multiple-choice questions, self-directed learning, learning analytics, engagement, expectancy value theory, strategic learning approach

## Abstract

This article was migrated. The article was marked as recommended.

**Background:** Multi-campus medical schools can differ in medical curriculum delivery due to location specific factors, creating different learning contexts. Common online learning may reduce perceived inequities. Using a shared curriculum structure, we developed two learning resource components (topic-based learning objects and multiple-choice question banks) in Obstetrics and Gynaecology for students in our 3-campus medical school.

**Objective:** We evaluated common learning resource use across different learning contexts. We hypothesised that students with fewer structured learning hours would make greater use of topic-based learning resources due to their perceived utility value. We also explored an alternative hypothesis; that resources more closely linked to assessment (MCQ banks) would encourage a strategic approach to learning and would be used most highly at all campuses.

**Methods:** We assessed student opinion of the value of the resources through a cross-campus online survey, and quantified usage of the resources by analysing learning management system logs. Comparisons of response and usage patterns for the two resource components were conducted to identify if context influenced usage.

**Results:** Survey results (RR = 70%) showed that students across campuses rated the resources as valuable. Usage logs partially supported our hypothesis that learning object usage would be highest at the campus (Campus 3) with the lowest structured learning hours in both the proportion of users (p <0.006) and frequency of access for 16 of the 26 topics (p<0.05). However, the reverse was found to be true for the question bank, with lowest usage of question banks at Campus 3 (p< 0.001).

**Conclusions:** We interpret the results as evidence of difference in the perceived utility value of the learning resources across campuses. Clear differences in usage patterns provide evidence that different learning contexts can influence online resource use, and these contexts should be considered when evaluating the effectiveness of online learning resources.

## Introduction

Medical schools differ widely in their structure and approach to curriculum delivery. While there are many commonalities within regions, internationally there is considerable diversity in approaches to medical education ( Boulet *et al.,* 2009). Factors such as population density and government funding affect the number and location of medical schools, and also shape the approach to curriculum delivery.

In New Zealand, there are two medical schools servicing a population of approximately 4.5 million people. The Otago Medical School (OMS) has, since the 1970s, had campuses for clinical teaching in three urban centres in New Zealand. These three campuses have evolved different approaches to curriculum delivery and different learning environments but have retained a common examination at the end of the second clinical year. This delivery model provides a natural laboratory for studying the impact of educational interventions.

In this structure, a discipline’s programmed curriculum time may differ at each campus. Because of the different exposure to programmed teaching in some areas of the curriculum some topics might receive less formal coverage, creating potential learning inequities for students that might not be recognised in the common assessment. Provision of common online learning resources is one way to address perceived inequities ( Ruiz, Mintzer and Leipzig, 2006).

Previous research ( Grant *et al.,* 2011; Ellaway *et al.,* 2014; Han, Nelson and Wetter, 2014) has identified that the uptake of online learning resources at different locations is dependent on contextual factors including; the perceived usefulness and value of the learning resources, the way these are promoted at each site, and the perceived alignment with the curriculum and assessment. Assessing the perceived value and usage of common learning resources at different campuses provides a mechanism for understanding how medical students’ engagement with learning resources is shaped by their learning contexts. One central question is whether usage is driven by the usefulness of the learning resources to broaden student learning about a discipline, or whether strategic choices are made which are driven by preparation for assessment. Theoretical approaches to both possibilities are expanded on below.

### Expectancy-value theory

The expectancy-value theory ( Eccles and Wigfield, 2002; Eccles, 2005) offers a method for understanding how students might perceive value in learning resources. Expectancy-value theory proposes that achievement-related choices are driven by two sets of self-perceptions, those which form a person’s expectations of their ability to succeed, and those that form perceptions related to the value of the task ( Eccles and Wigfield, 2002; Eccles, 2005, 2009; Brown *et al.,* 2015). In this model, the subjective task value is made up of several types of value that may be derived from the task being undertaken. These include interest value (how much enjoyment the task will give), attainment value (the value given to a task in terms of its ability to confirm characteristics central to a person’s identity), utility value (the value placed on the task as it relates to the ability to reach a goal, for example becoming competent in a discipline, or completing an assignment), and perceived cost (the cost of the task in terms of time, energy or its potential to impact identity) ( Eccles, 2005; Brown *et al.,* 2015).

Motivation to engage with learning opportunities in higher education students is particularly sensitive to utility value ( Brown *et al.,* 2015). Studies have found that utility value manipulations can be used to increase student interest in and persistence in tasks, even those which they previously found uninteresting or for which they had low expectations for success. For example, one study ( Hulleman *et al.,* 2010) found that encouraging students to make connections between course materials and their own lives increased student interest in and the perceived utility value of math, particularly for those students who previously had shown lower expectancies for success in math. Another study ( Hulleman and Harackiewicz, 2009) found that utility value and interest were increased when students were asked to complete a writing task to explain how they might or might not use the course material in their own lives. These studies indicate that utility value, which is strongly related to the relevance an activity holds for a person ( Brown *et al.,* 2015), can be influenced by making associations between activities and outcomes that are of value to the person.

### Strategic approaches to study

Other attempts to understand student motivation to engage with learning opportunities have focussed on identifying different approaches to learning ( Newble and Entwistle, 1986; Entwistle, 1988). Learning opportunities can be engaged with at surface or deep levels, and students can adopt a strategic approach to studying. The strategic approach is characterized by students allocating effort to learning based on their perception of its relevance to assessment ( Newble and Entwistle, 1986), a perception which might be influenced by judgements of a learning opportunity’s similarity to assessment (e.g. practice assessments) or an individual’s own perceived need to learn a topic in order to pass an assessment. Strategic approaches to learning are widely recognised in medical education. For example, one study found that when considering learning approaches of undergraduate and postgraduate medical students, undergraduate students were more likely to employ strategic learning approaches in their study ( Samarakoon *et al.,* 2013). A study by Reid and colleagues ( Reid, Duvall and Evans, 2009) found that even when a course is purposefully designed to promote deep learning, students tend to show high levels of strategic learning approaches alongside deep learning approaches. In their discussion they speculated that this may be due to students continuing to use the approach that has worked for them in the past.

Previous research investigating the uptake of online medical education resources in different sites, adopted a qualitative research methodology to understand the factors that influenced usage of these resources ( Ellaway *et al.,* 2014). Interviews with residents completing surgery clerkships suggested that decisions to engage with online learning resources were influenced by both perceptions of utility value and application of strategic learning approaches. That is, interviewees reported using online learning resources more when they were seen to be good preparation for examination, supported them in preparing for workplace encounters with patients, or were determined to be high value based on feedback from other users. Students also reported using alternative learning resources if they were considered more useful than the online learning resources that were being provided in the study. However, this study does not include an analysis of actual usage across sites to measure possible variation. Analysis of usage data from online delivery systems is one means of obtaining objective data to compare with the self-reported attitudes that are revealed through survey and interviews ( Bientzle *et al.,* 2019), and provides a mechanism for identifying patterns of usage that could provide evidence of differences in utility value and strategic learning in different learning contexts.

### The present study

This paper presents an investigation into the usage, reported and actual, of online learning resources - consisting of topic-based learning objects and a common multiple-choice question (MCQ) bank - that were developed to support self-directed learning in Obstetrics and Gynaecology (O&G) at three campuses. The topic-based learning resources are designed to support understanding of core concepts in O&G, while the common MCQ is clearly identified as a mechanism for learning and assessment preparation, with students being explicitly informed that all MCQ questions in the O&G component of the end of module assessment would be drawn from this bank. For this reason, usage of the MCQ bank would support a strategic approach to study due to its explicit link to assessment. Usage of the MCQ bank and topic-based learning resources may also be driven by perceptions of utility value, which may differ by campus. The three campuses differ in the amount of time allocated to formal learning in O&G, with Campus 3 experiencing less formal contact time in this discipline than Campuses 1 and 2. This allows us to investigate the extent to which the usage of the two types of learning resources might be subject to differences in perceived utility value, or if strategic approaches to learning for assessment over-ride the broader achievement-based goals of students to understand the discipline.

Education research conducted in real-life contexts can be considered similar to effectiveness studies ( Flay *et al.,* 2005; Ellaway *et al.,* 2014), which accept a reduction in experimental control of variables that might influence the impact of an intervention in favour of investigating the performance of an intervention in real-world conditions ( Flay *et al.,* 2005). In doing this, it is accepted that site-specific factors may affect the success of an intervention, which may become apparent in both self-reported evaluation by users and in their actual usage data. Ellaway and colleagues ( Ellaway *et al.,* 2014) identified a number of these factors in their work, including presence or absence of scheduled curriculum time for use of learning resources, assessment incentives, and mandatory use of some modules. In our study, assessment incentives for the MCQ bank were common for all campuses, but Campus 3 had an additional instruction to review a small number of the total set of topic-based learning resources. This instruction may influence the perceived utility value of these resources, which might be detected in higher usage of the specified resources than other topic-based resources. Furthermore, it and other site-specific differences such as reduced curriculum time for the discipline at Campus 3 might generate differences in opinions of the relative value of both the MCQ bank and topic-based resources, which may also be revealed in differences in patterns of usage between the campuses.

The aim of this study was to investigate the impact of site-specific factors on self-reported value and usage of online learning resources. Our awareness of some differences in Campus 3 allow us to make two hypotheses that, if supported, would provide evidence for differences in utility value between resource types. We may also see evidence of strategic approaches to learning. We hypothesised that students who had fewer hours of formal teaching (Campus 3) would perceive the topic-based learning resources as having higher utility value than the other campuses, and demonstrate higher usage and higher reported value than their counterparts at other campuses where more formalised teaching occurs. Assuming that the instruction for mandatory use of five topics at Campus 3 generates higher perceived utility value for these resources, we also hypothesise that usage for these five topics would be higher than usage of other topic-based learning resources for students at this campus. An alternative hypothesis would be that students are motivated solely by strategic study approaches, and show an overall tendency to favour the use of the MCQ bank in preference to the topic-based learning resources.

## Methods

To investigate the contextual factors that might influence uptake, we conducted an online survey with students from each campus to determine if there were differences at each campus. The survey measured the perceived value of the learning resources and the extent to which users found them engaging. We then conducted an analysis of usage logs in the OMS Learning Management System (Moodle) for the three campuses to investigate the overall uptake and objective patterns of usage of the resources, and determine if there were differences in usage.

### Survey of perceived value of online resources

Sampling of a representative proportion of students and using personalised invitations and intensive follow-up procedures ( Dillman, Smith and Christian, 2014) was chosen to generate a high sample response rate. A random sample of 60% of eligible students was drawn independently from each campus, and deemed to be a practical number of potential respondents to manage a personal follow up process if needed. To be eligible for inclusion, students had to have either begun or completed their O&G attachment at the time of the survey. These selected students were sent a personalised hard-copy letter inviting them to participate in a survey along with an information sheet about the study and a pen. Following this, an email invitation was sent containing a link to the survey. Participants indicated informed consent by clicking on the emailed link to complete the survey. A follow up email to non-respondents was sent one week later.

The survey was conducted using SurveyMonkey (SurveyMonkey Inc., 2020). Respondents were asked to identify their campus, and the learning activities they had accessed - the topic-based Learning Objects, the Common MCQ bank, or “neither”. Respondents were then asked a further 10 questions to measure engagement with the online learning resources (adapted from ( Gallagher, 2015)) using a 5-point Likert-type scale from “strongly disagree” (1) to “strongly agree” (5). Two additional items asked respondents to rate the challenge of the learning objects and the common MCQ bank separately, on a 5-point scale where 1 = “Far too simple”, 3 = “An appropriate level of challenge”, and 5 = “Far too advanced”. Differences in the ratings on these items were assessed using a Kruskal-Wallis test. Respondents were also invited to comment on the most valuable and least valuable aspects of the on-line resources.

### Analysis of Moodle logs

Logs for all the students in the three campuses who used the resources were exported from Moodle and analysed using Stata 15.1 ( StataCorp, 2017). The date range for the logs was selected to begin when students were first given access to the online resources (3 ^rd^ February 2015), and to end when the common examination occurred (2 ^nd^ November 2015). Logs for the use of the learning resources and the common MCQ bank were analysed separately.

The logged data were reviewed by three authors (SG, SH and TG), and consensus was reached regarding events that could be removed from analysis due to their overlap with other events in the logs. Events in logs relating to activities and resources were grouped according to their topic in order to determine if there were different patterns of usage for topics across the three campuses.

To assess the usage by all students of each of the 26 sets of topic-based learning objects as well as of the MCQs, a summary measure of activity for each learning object was calculated by summing the number of logged events associated with that topic. For comparison between campuses, the number of unique users who accessed a resource was expressed as a proportion of the total number of potential users. Pearson’s Chi-squared test was then used to assess any observed difference in the proportion of users of each topic from the expected distribution.

To assess if there was a difference in the frequency of usage between the three campuses, the number of times resources in each topic were accessed was summarised by calculating the median and inter-quartile range for each campus. Kruskal-Wallis tests were conducted to identify topics where frequency of use differed by campus. For topics with a significant difference, Dunn’s pairwise comparison tests were conducted to pinpoint where the difference occurred.

## Results/Analysis

### Online survey

The range of response rates across campuses was 66% to 73%, with an average response rate of 70%. Table 1 shows the total number of students, eligible students, the number selected and the response rates for each campus. Across the three campuses, 63% of respondents reported accessing the learning objects, and all respondents reported accessing the MCQ bank.

**Table 1:** Sampling and response rates by campus

Campus	Total number of students	Number of students meeting eligibility criteria	Number selected	Response rate
Campus 1	107	84	50	66%
Campus 2	107	74	45	71%
Campus 3	79	55	33	73%

The median and IQRs for the responses to the 10 questions about the learning resources indicate favourable responses, with each question have a median score between 3 (neither agree nor disagree) and 5 (strongly agree). The ratings were not significantly different between campuses when evaluated using the Kruskal-Wallis test, with the exception of one item “The material was relevant to my level of learning” ( *p*= 0.005): the means calculated for this item showed Campus 3 had a lower mean rating (3.65) than Campus 2 (4.30) and Campus 1 (4.26).

The challenge rating for the learning objects did not differ between campuses, with each campus having a median rating of 3 or “An appropriate level of challenge” ( *p*=0.0587). The challenge rating for the MCQ bank was also not significantly different ( *p*=0.0697 *)* between campuses, although Campus 2 reported a greater level of challenge for the MCQs (median = 4, “More difficult than anticipated”) than the other two campuses (median = 3).

### Analysis of Moodle logs

During the period of the study, 88% of possible users at Campus 1, 77% of possible users in Campus 2, and 99% of users at Campus 3 accessed at least one learning object resource. All users at Campus 2, and all but one user at Campus 1 and at Campus 3 accessed at least one quiz in the MCQ bank. A higher percentage of users accessed the question bank when compared to the learning objects at Campuses 1 and 2, while at Campus 3 the two components were accessed by the same percentage of users.

### Topic-based learning resource analysis

Usage analysis for each topic-based learning resource found the proportion of users who accessed the topics was significantly higher (p <0.006) at Campus 3 for all topics. At Campus 3 the least popular topic was accessed by 22% of students, and the most popular topic was accessed by 97% of students. The least popular topics for Campus 1 and 2 were accessed by 5% and 7% of students respectively, and the most popular topics for Campus 1 and 2 were accessed by 88% and 77% of students respectively.

The median number of times that a topic was accessed was calculated to summarise differences in rates of usage. These are shown in Table 2 for each topic by campus. Sixteen of the 26 topics had differences in the frequency of use at the 5% level of significance. Of those 16 topics, Campus 3 had a significantly higher frequency of access than either one or both of the other campuses.

**Table 2:** Median frequency of access to learning objects by students in each campus (with inter-quartile range)

	Campus 1	Campus 2	Campus 3	Kruskal-Wallis
Topic	Med (IQR)	Med (IQR)	Med (IQR)	p-value
Topic 1 *^a^*	6 (6)	4 (5)	7 (7)	< 0.001
Topic 2	2 (2)	2 (1)	3 (2)	0.279
Topic 3 *^b^*	8 (17)	6 (11)	36 (45)	< 0.001
Topic 4 *^a^*	4 (3)	2 (3)	4 (4)	0.027
Topic 5	8 (7)	5.5 (6.5)	8 (9)	0.106
Topic 6 *^b^*	6.5 (6.5)	4 (5)	9 (8)	0.012
Topic 7	5.5 (5)	6 (5)	6.5 (6)	0.536
Topic 8	8.5 (6)	8 (9)	8 (16)	0.765
Topic 9	2 (0)	2 (1)	2 (1)	0.819
Topic 10 *^a^*	6 (6)	4 (6)	7.5 (7.5)	0.044
Topic 11 *^a^*	1 (3)	1 (1)	3 (1)	0.012
Topic 12	5 (4)	8 (5)	8 (9)	0.086
Topic 13	4 (4)	4 (3)	6 (5)	0.491
Topic 14	6 (5)	4.5 (6)	6 (12)	0.310
Topic 15	2 (3)	4 (3)	4.5 (8.5)	0.055
*Topic 16 *^b^*	7.5 (8)	5.5 (5)	13 (18.5)	<0.001
Topic 17 *^b^*	3 (2)	4 (2)	9 (7.5)	<0.001
Topic 18 *^a^*	15 (14)	5.5 (4.5)	17 (19)	0.010
Topic 19 *^b^*	5 (12)	2 (6)	11 (18)	<0.001
Topic 20	9.5 (14.5)	4 (4)	10 (25)	0.382
*Topic 21 *^b^*	3 (4)	4 (4)	12 (13)	<0.001
*Topic 22 *^b^*	6 (5)	4 (2)	9 (8)	0.005
*Topic 23 *^b^*	3 (3)	4 (2.5)	10 (10)	<0.001
Topic 24 *^a^*	4 (1)	4 (1)	7.5 (5)	0.073
Topic 25 *^a^*	4 (13)	3 (1)	11 (15)	0.010
*Topic 26 *^b^*	5 (4)	4 (2)	7 (7)	0.005

^a^ Indicates topics where access was significantly more frequent at Campus 3 than either Campus 1 or Campus 2.

^b^ Indicates topics where access was significantly more frequent at Campus 3 than at both other campuses.

* Recommended Topics for students at Campus 3.

### MCQ bank analysis

The proportion of users who accessed each quiz ranged between 0.79 and 0.89 for Campus 2 students, 0.72 and 0.88 for Campus 1 and 0.52 and 0.80 for Campus 3. In all cases, Campus 3 had the lowest proportion of student users accessing each MCQ topic, and in 21 of the 26 quizzes, this difference was significant (p< 0.001) when compared with Campus 1 and Campus 2.

The frequency of access was analysed by calculating the median number of times each quiz was accessed and the inter-quartile range ( Table 3). Quizzes were aligned with the same topics as the learning object, and in some cases more than one quiz was associated with a topic. This resulted in a total of 48 individual quizzes. There were no significant differences in the frequency of use by students at the three campuses in 45 of the 48 quizzes. Three quizzes had differences in the frequency of usage across campuses at the 5% level of significance. A 49 ^th^ quiz incorporated all questions in the question bank. This quiz also showed significant differences in usage between campuses. In each case of a significant difference, Campus 3 exhibited lower usage that either one or both of the other campuses.

**Table 3:** Median frequency of access to quizzes by campus (with inter-quartile range)

	Campus 1	Campus 2	Campus 3	Kruskal-Wallis
Quiz	Med (IQR)	Med (IQR)	Med (IQR)	p-value
Quiz 1	11 (10)	12 (10)	10 (9.5)	0.1558
Quiz 2	10.5 (9)	11 (10)	10 (9)	0.5223
Quiz 3-1	10 (11.5)	10 (11)	8 (7)	0.2966
Quiz 3-2	6 (5)	8 (8)	6 (6)	0.0517
Quiz 4 *^b^*	10 (10)	9.5 (9)	7 (6)	0.0092
Quiz 5-1	7 (5)	8 (6)	7 (8)	0.7871
Quiz 5-2	7 (7)	10 (7.5)	6 (6.5)	0.0898
Quiz 5-3	6 (7)	7 (8)	6 (5)	0.7149
Quiz 6	8 (9.5)	8 (8)	8 (7)	0.9257
Quiz 7-1	9 (8)	11 (9)	7 (8)	0.4437
Quiz 7-2	7.5 (8)	8 (10)	7 (8)	0.3105
Quiz 7-3	8.5 (7.5)	8 (9)	7 (5)	0.4074
Quiz 8-1	7 (6)	7.5 (8)	7 (6)	0.434
Quiz 8-2	7 (6)	8 (11)	6.5 (6)	0.6119
Quiz 9	6 (6)	9 (6)	7 (7)	0.0843
Quiz 10 *^b^*	7 (10)	7 (8)	6 (5)	0.0237
Quiz 11-1	7 (7)	10 (8)	6 (6)	0.2222
Quiz 11-2	6 (8)	8 (8)	6 (7)	0.4282
Quiz 11-3	7 (6)	7 (8)	6 (5)	0.132
Quiz 12	6 (7)	8 (9.5)	6 (7)	0.09
Quiz 13-1	8.5 (9)	8 (9)	6.5 (6)	0.2754
Quiz 13-2	8 (9)	7 (8)	7 (6)	0.2025
Quiz 14	6 (7)	7 (7)	6.5 (6)	0.7592
Quiz 15-1	7 (7)	7 (7)	6 (6.5)	0.599
Quiz 15-2	10 (6)	10 (8)	6.5 (7)	0.4422
Quiz 16-1	7 (7)	6 (7)	7 (5)	0.8862
Quiz 16-2	7 (5)	6 (6)	6 (3)	0.0986
Quiz 17	7 (7)	8 (6)	7 (5)	0.7399
Quiz 18	6 (6)	7 (7)	7 (6)	0.4664
Quiz 19-1 *^a^*	13 (13)	8 (10)	8 (7)	0.001
Quiz 19-2	7 (8)	6 (7)	6 (5)	0.2184
Quiz 19-3	7 (6)	6 (6)	6 (2)	0.3829
Quiz 20-1	7 (6)	6 (6)	6 (5)	0.2714
Quiz 20-2	6 (6)	6 (6)	6 (5)	0.6099
Quiz 20-3	7.5 (7)	6 (6)	6 (5)	0.1569
Quiz 20-1	6 (6)	6 (6)	6 (4)	0.5618
Quiz 20-2	6 (2)	6 (5)	5 (1)	0.1275
Quiz 21-1	6.5 (7)	6 (6)	6 (6)	0.3672
Quiz 21-2	7 (6)	6 (5)	6 (2.5)	0.1296
Quiz 21-3	6 (7)	6 (6)	6 (6)	0.6588
Quiz 22	7 (7)	6 (6)	7 (6)	0.3899
Quiz 23-1	7 (7)	6 (6)	7 (5)	0.5309
Quiz 23-2	6 (6)	6 (6)	6 (5)	0.9629
Quiz 23-3	6 (6)	6 (6)	6 (5)	0.8968
Quiz 24	6 (6)	6 (6)	6 (3)	0.6897
Quiz 25-1	6 (6)	6 (6)	6 (3)	0.5348
Quiz 25-2	6 (6)	6 (6)	6 (2)	0.6244
Quiz 26	6 (6)	6 (7)	7 (6)	0.7932
Quiz : Whole Bank ^a^	13 (41.5)	6 (16.5)	6.5 (13)	0.0028

Quizzes are numbered to indicate the topic that they relate to using the same numbering system as the analysis of topic-based learning resources. Hyphenated numbers indicate more than one quiz was associated with a topic.

^a^ Indicates quizzes where access was significantly less frequent at Campus 3 than either Campus 1 or Campus 2.

^b^ Indicates topics where access was significantly less frequent at Campus 3 than at both other campuses.

### Discussion

There is clear evidence that students at Campus 3 demonstrated different learning resource usage patterns than Campuses 1 and 2. Students at Campus 3 demonstrated higher levels of usage of the topic-based learning resources, both in terms of the proportion of students who accessed these resources and in terms of the frequency with which the resources were accessed. However, fewer students at Campus 3 engaged with the MCQ question than the other campuses. For those students who did engage with the MCQ bank from Campus 3, their frequency of usage was not significantly different except for four quizzes. The evaluation survey assessed student’s self-reported engagement with the online learning resources, and included items that assessed multiple dimensions of engagement, specifically; access, navigation, motivation, relevance, duration, flexibility, support, endurability, integration, presentation and challenge ( Gallagher, 2015). Student ratings were positive and, with the exception of an item relating to relevance that was rated lower by Campus 3 students, did not differ between campuses. The survey results suggest that the students found the online learning resources to be engaging and at an appropriate level of challenge. On this basis, we cannot explain differences in the usage of the resources at Campus 3 as a result of differential engagement at the three sites.

It is possible that the differences observed related to differences in the perceived utility value of some resources. Students at Campus 3 used more of the learning objects than their peers at the other campuses when measured as a proportion of students accessing the resources. Students at Campus 3 also used the learning resources more frequently than the students at the other campuses. Although Campus 3 students were instructed to review 5 specific topics, and the usage of these topics is more frequent for students at Campus 3, they also displayed higher frequency of use than at least one other campus in 11 additional topics. Differential instructions are recognised as a potential cause of differences in usage ( Ellaway *et al.,* 2014), and might be considered to drive the increased use of topic-based learning resources at this campus; however, we also observed an overall lower level of usage of the MCQ question banks at this campus. Our hypothesis that these instructions would serve as a utility value intervention and result in higher usage for these five topics is partially supported, however the higher usage at Campus 3 is not restricted to these topics. We therefore interpret the finding of higher overall usage of the topic-based learning resources by Campus 3 students as evidence of an overall higher perceived utility value for these resources due to the difference in learning context. The instruction to these students may have influenced the perception of utility value of the topic-based resources. We also recognise that students at all three campuses clearly used the MCQ banks, though at different rates, and can see this as evidence of both a desire to diagnose their levels of knowledge and to take advantage of the strategic benefit of practicing assessment. We find it interesting, however, that the desire to learn topics well may have trumped the strategic value of assessment practice at Campus 3.

These findings may be understood through differences in the perceived utility value between Campus 3 and the other campuses. We might infer that the topic-based learning resources were more highly valued at Campus 3 due to the more limited time allocated to formal teaching, and that spending time revising these resources was deemed to have more utility value than practicing the MCQs. It is also possible that students at Campus 3 found more utility value in the use of these resources as the instruction to access these topics might be perceived as an indication that they would be useful for their further study. In contrast, students at Campuses 1 and 2 perhaps felt they had sufficient formal teaching to cover the topics adequately, and therefore exhibited a strategic approach to learning, and showed preference for the MCQ question bank in line with its alignment with summative assessment.

Given the previous finding in multi-site studies of online learning resources ( Ellaway *et al.,* 2014) that alignment with assessment is a significant contributor to perceived value, this is not surprising. However, our analysis of usage data suggests that in some contexts learning resources may attract higher usage than resources that are explicitly linked with summative assessment. This finding fits well with expectancy value theory, which posits individual perceptions of the different value types will determine how much time and energy is spent on different types of tasks ( Eccles, 2005; Brown *et al.,* 2015). We might therefore infer that the topic-based learning resources were more highly used at Campus 3 as preparation for summative assessment, and that this was valued more highly than MCQ practice for the assessment at Campus 3. Overall, this supports the framework presented by Ellaway and colleagues ( Ellaway *et al.,* 2014) that highlights the multi-factorial context that influences uptake of learning resources.

The open design of the resources; which supported student choice about which resources they accessed, and in which order; mitigated against issues with student agency, an important factor in Ellaway *et al’s* (2014) model. A lack of student agency, where there is limited control over the way in which students engage with online resources, is identified as problematic for uptake of online resources.

This study demonstrates the value of taking a comprehensive approach to the evaluation of education initiatives. We showed that usage data is important to incorporate as a triangulation method in evaluation to investigate student learning patterns. Through the use of both reported and objective data collection methods across three campuses, this study allowed us to identify that the uptake of the two components of the learning resources differs across settings. Rather than concluding that one type of learning resource is superior to the other, this study demonstrates the importance of providing students with the ability to choose the learning support that is most helpful in their context.

### Limitations

Despite the comprehensive approach taken, there are some limitations in the study that restrict the generalisability of the findings. As a cross-sectional study, it provides only a snapshot of usage and does not allow us to assess the longevity of the effects. It may be that the novelty of such a structured and thorough set of complementary learning resources stimulated use and influenced self-reported ratings of the value of the resources. There is also a potential limitation in the directed nature of usage for Campus 3 compared to Campus 1 and 2. However, our analysis of usage makes it clear that the direction to use a small number of topics did not increase usage for those topics only, and suggests that either this served as a utility value intervention that influenced use of all topics, or that there was a context-specific difference in utility value associated with the topic-based learning resources at this campus. Despite these limitations, this study has demonstrated that the provision of online resources to support learning in a three-campus medical school is seen positively by students, and that differences in patterns of usage can emerge in response to the local learning context.

## Conclusion

This study identified one mechanism for reducing potential learning inequities that can occur in multi-site medical schools; that is, providing common learning resources that are accessible regardless of the student’s physical location. The value of detailed analysis of usage, alongside self-reported evaluation, has been demonstrated as a way of assessing different patterns of learning and as a source of evidence of strategic learning techniques in response to local learning environments. This study provides objective evidence that, when evaluating the effectiveness of interventions to address potential inequities in learning opportunities, it is important to consider attitudes and behaviour (usage) at all sites where differences in learning context may be expected. Had we relied solely on the opinions gathered in our online survey, or looked at behaviour at only one site, we would not have appreciated the differences that can occur in perceived utility value and usage at sites with different learning contexts.

## Take Home Messages

• Students will strategically allocate time to resources that are perceived to have a high value in their particular learning context
• Usage logs for online learning are an important source of data for understanding the influence of local factors on the uptake of online resources
• Students feel a greater sense of agency when given the ability to determine how, when, and where they use learning resources
• Cross-campus collaboration and resource sharing can offer students a more equitable learning experience even when they are based in multiple campuses across geographic locations

## Notes On Contributors

Steve Gallagher (PhD) is a lecturer in eLearning at the Otago Medical School - Dunedin Campus, with a background in behavioural psychology and an interest in learning technology and self-efficacy. ORCiD: https://orcid.org/0000-0003-1108-4388

Tehmina Gladman (PhD) is a lecturer and Education Adviser at University of Otago, Wellington. Her background is in experimental psychology and her research interests include academic motivation and motivation to use technology for learning. ORCiD: https://orcid.org/0000-0002-5112-3460

Emma Macfarlane (MHealthSci) is a Nurse Practitioner working and teaching in reproductive and sexual health care at the Otago Medical School - Dunedin Campus. ORCiD: https://orcid.org/0000-0001-6725-1224

Scott Hallman is an eLearning Facilitator at the University of Otago, Christchurch.

John Hutton is a Professor in the Department of Obstetrics and Gynaecology at the Wellington School of Medicine and Health Sciences in the University of Otago. In the last 10 years, his academic focus has been on the development of different e-learning resources for undergraduate students of obstetrics and gynaecology.

Helen Paterson (PhD, FRANZCOG) is an academic gynaecologist in the Department of Womens’ and Childrens’ Health at the Dunedin School of Medicine. ORCiD: https://orcid.org/0000-0002-8240-082X
