# Supplementary material for: Student use of common online resources in a multi-campus medical school
Source: MedEdPublish (2016). 2021 Feb 26;10:56. [Version 1] doi: 10.15694/mep.2021.000056.1 (PMC10939622; doi:10.15694/mep.2021.000056.1)
Supplement: Peer review history

# Reviewer report: Reviewer response for version 1

**DOI:** 10.21956/mep.18956.r27133

**Published:** 2021-06-07

**Authors:** Ken Masters

**Affiliations:** 1 Sultan Qaboos University

This review has been migrated. The reviewer awarded 3 stars out of 5 An interesting paper on student use of common online resources in a multicampusmedical school.The setting is a rather unconventional one, with a single university spread across three campuses that appear to have quite a bit of independence in their teaching approaches, but have a common examination. The aim is to assess the extent to which contextual factors of each campus may impact on students’ use and perceptions of value of their university’s online resources. Although the setting is localised, the common curriculum with the variety of methods does provide a useful method for comparing different groups of students’ perceptions.The study is well-grounded in the theory, and well-conducted.Issues that need to be resolved are mostly in the presentation of the paper:• More detail is required on the design of the questionnaire. The dates of the survey should also be given.• It would also be useful if the actual questionnaire were to be given (as a supplementary file or appendix).• Given that there were only 10 questions, it would be useful if the stats (showing the number and percentage) of students’ selection for each question, per campus) for all 10 questions could be given in a table, rather than the few lines currently given.• In all the text (and tables) both raw numbers and percentages (not percentages only) should be given.• Table 2: A supplementary file, identifying each topic would be useful. • As we do not have access to the questionnaire, we do not know whether or not demographic data (especially age and gender) were gathered. As studies have shown the impact of these on online work:o If they were not gathered, the authors should explain why not.o If they were gathered, then readers should be shown a breakdown of data along these lines, and the authors should perform statistical tests to see if there were any significant differences. Minor:• “with each question have a median” I think the “have” should be “having”.So, overall a useful read, but some more detail is still required. I look forward to Version 2 of the paper in which these issues are addressed.

Reviewer Expertise:

NA

No decision status is available

# Reviewer report: Reviewer response for version 1

**DOI:** 10.21956/mep.18956.r27132

**Published:** 2021-02-27

**Authors:** P Ravi Shankar

**Affiliations:** 1 American International Medical University

This review has been migrated. The reviewer awarded 4 stars out of 5 Thank you for the invitation to review this interesting and well-presented manuscript. Multi-campus medical schools are not common in most parts of the world, though a few organizations are working toward setting up campuses at multiple locations and ensuring standardized delivery of teaching-learning and course materials and assessment across sites. The Otago Medical School according to the description in the manuscript pursues different curricula and teaching-learning activities across the three campuses. I am interested in knowing why this model was chosen instead of having the three campuses as separate medical schools. In Australia, Canada and I assume also New Zealand education in rural healthcare facilities is considered very important and students spend considerable time in these locations. From the reference to the Moodle logs description, my understanding is that the study was conducted in 2015 and there is a lag period between the study conduct and the publication. In the interim several changes have occurred including the coronavirus pandemic which has given a major fillip to online learning. What type of topic-based learning resources were created? Did these use videos or web-based clinical scenarios? Were all students accessing the resources from cities and towns? Did any students access these resources from rural areas? Were there any internet bandwidth issues?I did not fully understand about the sampling method used in this study. Why were 60% of eligible students chosen? I like the idea of sending the students a hard copy letter along with a pen before sending them the email invitation. The authors have not mentioned about ethical approval or exemption for the present study. If the study was done in 2015 them some of the circumstances may have changed and this should be considered while considering the findings.

Reviewer Expertise:

NA

No decision status is available
